# Antibacterial Activity of Silver and Gold Particles Formed on Titania Thin Films

**DOI:** 10.3390/nano12071190

**Published:** 2022-04-02

**Authors:** Mantas Sriubas, Kristina Bockute, Paulius Palevicius, Marius Kaminskas, Zilvinas Rinkevicius, Minvydas Ragulskis, Sandrita Simonyte, Modestas Ruzauskas, Giedrius Laukaitis

**Affiliations:** 1 Physics Department, Kaunas University of Technology, Studentu Str. 50, LT-51368 Kaunas, Lithuania; mantas.sriubas@ktu.lt (M.S.); marius.kaminskas@ktu.lt (M.K.); giedrius.laukaitis@ktu.lt (G.L.); 2 Department of Mathematical Modeling, Kaunas University of Technology, Studentu Str. 50, LT-51368 Kaunas, Lithuania; paulius.palevicius@ktu.lt (P.P.); minvydas.ragulskis@ktu.lt (M.R.); 3 Division of Theoretical Chemistry & Biology, KTH Royal Institute of Technology, School of Biotechnology, 109 61 Stockholm, Sweden; rinkevic@kth.se; 4 Institute of Microbiology and Virology, Faculty of Veterinary Medicine, Veterinary Academy, Lithuanian University of Health Sciences, Tilzes Str. 18, LT-47181 Kaunas, Lithuania; sandrita.simonyte@lsmuni.lt (S.S.); modestas.ruzauskas@lsmuni.lt (M.R.); 5 Institute of Cardiology, Medical Academy, Lithuanian University of Health Sciences, Sukileliu Ave. 15, LT-50162 Kaunas, Lithuania; 6 Department of Anatomy and Physiology, Lithuanian University of Health Sciences, Tilzes Str. 18, LT-47181 Kaunas, Lithuania

**Keywords:** TiO_2_ thin films, silver nanoparticles, gold nanoparticles, Gram-negative bacteria

## Abstract

Metal-based nanoparticles with antimicrobial activity are gaining a lot of attention in recent years due to the increased antibiotics resistance. The development and the pathogenesis of oral diseases are usually associated with the formation of bacteria biofilms on the surfaces; therefore, it is crucial to investigate the materials and their properties that would reduce bacterial attachment and biofilm formation. This work provides a systematic investigation of the physical-chemical properties and the antibacterial activity of TiO_2_ thin films decorated by Ag and Au nanoparticles (NP) against *Veillonella parvula* and *Neisseria sicca* species associated with oral diseases. TiO_2_ thin films were formed using reactive magnetron sputtering by obtaining as-deposited amorphous and crystalline TiO_2_ thin films after annealing. Au and Ag NP were formed using a two-step process: magnetron sputtering of thin metal films and solid-state dewetting. The surface properties and crystallographic nature of TiO_2_/NP structures were investigated by SEM, XPS, XRD, and optical microscopy. It was found that the higher thickness of Au and Ag thin films results in the formation of the enlarged NPs and increased distance between them, influencing the antibacterial activity of the formed structures. TiO_2_ surface with AgNP exhibited higher antibacterial efficiency than Au nanostructured titania surfaces and effectively reduced the concentration of the bacteria. The process of the observation and identification of the presence of bacteria using the deep learning technique was realized.

## 1. Introduction

A majority of bacteria live in communities that are associated with surfaces, and this property is considered as the most essential of bacteria attachment and colonization in different environments [1]. An organized bacterial community attached to the surface exists in biofilms, which by different bacterial species are associated with many human illnesses and infections, such as oral diseases [2]. Bacteria have a self-produced extracellular matrix that is detected in almost all biofilms [3,4]. The majority of cariogenic oral biofilm matrixes contain exopolysaccharides (EPS) as the main components, and they are considered as virulence factors in the development and pathogenesis of oral diseases, such as caries, gingivitis, and periodontitis [5]. EPS provides protection from mechanical damage and shear caused by fluid flow but also protects bacteria from the host immune system and antimicrobial agents [1,6].

Oral diseases related to cariogenic oral biofilms, such as dental caries, affect people of all ages worldwide [5]. Human dental plaque is a well-recognized example of a natural biofilm that plays an important role in the pathogenesis of oral diseases. Studies have shown that oral bacteria that are involved in the development of the periodontal disease can move into the bloodstream and increase the risk of cardiovascular disease [7,8]. It is known that oral biofilms often exhibit resistance to antimicrobial treatment and host immune responses, thus becoming persistent colonizers or sources of chronic infections [1,9]. The *Veillonella* species, along with the *Neisseria* species, were found to be consistently resistant to tetracycline, penicillin, and ampicillin. The genus *Veillonella* and *Neisseria* consist of small, non-spore-forming, Gram-negative diplococci [10]. As strictly anaerobic *Veillonella* is a regular component of supragingival dental plaque and normal microbiota of the tongue, while obligate aerobe *Neisseria* colonizes human mucosal epithelia; however, at different sites in the body. The species of *Veillonella* and *Neisseria* genera have cellular-surface molecules, including membrane proteins such as pili, capsule, and lipooligosaccharide involved in the formation of biofilm [9]. It is known that *Neisseria* motility is enabled by pili which also mediates an attachment to host cells, colonization, and biofilm formation [11].

Bacterial cell surface properties and physical-chemical properties of the surface such as topography, roughness, and surface charge are essential for bacteria attachment and biofilm formation [12]. Previous research has established the influence of the different surface topography and roughness on microbial adhesion and bacteria attachment inhibition by reducing the contact area between bacteria cells and the surface [13,14,15,16,17]. It has been observed that the surface roughness can increase the surface area and therefore decrease bacteria attachment and colonization at a micrometer scale [18,19,20], and the increased surface roughness can decrease the number of adhesion points and reduce attachment area between the cells and the material at a nanometer scale [15,16,17].

Titanium (Ti) and its alloys are the most commonly used materials for orthopedic and dental implants due to their good mechanical properties, corrosion resistance, and biocompatibility [21]. Additionally, titanium dioxide is a well-known nontoxic photocatalytic material that can perform as an antimicrobial agent due to the photocatalytic processes occurring on the surface of TiO_2_ when irradiated with UV light [22,23]. However, biofilm formation remains a critical factor for implant-associated infections which are the leading cause of implant failure [24,25]. Implants coated with antimicrobial agents such as antibiotics prevent bacteria attachment and biofilm formation, but this approach is usually effective in short-term applications as prolonged use may cause drug resistance [26,27]. As an alternative, nanoparticles, such as silver (Ag), gold (Au), and copper (Cu) have been demonstrated to have a broad-spectrum bactericidal or bacteriostatic activity at low concentrations without producing resistant bacteria [28,29,30]. Studies have shown that Ti and TiO_2_ surface modifications and thin films with metal nanoparticles have a similar effect as antibiotics on bacterial adhesion, colonization, and biofilm formation [12,20,31,32,33]. Although the use of nanomaterials decreased with the prevalence of antibiotics, compared to organic agents they have chemical stability and thermal resistance thus used to create unique surfaces with altered physical and chemical characteristics [34,35]. A number of different antibacterial mechanisms of action for nanoparticles as an antibacterial agent are described [36,37,38]. For example, it has been observed that AgNPs inhibit DNA replication, damage the cell membrane, and inactivate proteins. They also affect the purine metabolite pathway, make penetration through cell membrane easier, cooperate with intracellular materials, and allow cell destruction. While AuNPs are not limited to reactive oxygen species (ROS) activity. They can target the energy metabolism, transcription process of bacteria, and cross the bacterial surface to form an abnormal subcellular spherical cytoplasmic structure called an inclusion body of gold nanoparticles (IB-AuNPs), leading to disruption of the bacterial cell membrane and the death of bacteria [39,40]. However, the key antimicrobial efficiency points are related to adhesion to the cell membrane, intracellular penetration, generation of reactive oxygen species (ROS), and toxic effects on intracellular processes [41]. Therefore, the additional incorporation of an inorganic antimicrobial agent of Ti surface that would allow having various molecular mechanisms of antimicrobial activity could be used as an effective approach for preventing biofilm formation and biomaterial-associated infections, but also to fight the growing number of multi-resistant bacteria. However, not all studies found antimicrobial efficiency of nanomaterials in comparison to untreated titanium, thus there is still a need for further studies to design better antibacterial biomaterials.

E-beam lithography [42], nanoimprint lithography [43], and chemical synthesis [44] are extensively used for the fabrication of metal nanostructures. However, these methods are time-consuming, expensive, and have a low fabrication yield [4]. One of the simplest and fastest methods for the formation of nanoparticles (NP) is a solid-state dewetting of thin metallic films. The as-deposited films are metastable or unstable and transform into smaller structures such as islands, droplets, or particles following the surface energy minimization at high enough temperatures below the melting temperature [45]. Pre-existing defects, such as holes, grain boundaries, and thin film edges, are common in thin films and act as the initiation sites for the dewetting process. The process is composed of several steps including edge retraction, pinch-off, fingering, and Rayleigh instability [46]. Thin films start to form a rim at the grain boundaries or edge of the film when they are heated, then the mass diffuses from the triple point, creating a hole and the rim on the film surface (edge retraction). The holes expand and a thickened surface at the edge of the hole develops. Simultaneously, the valley behind the rim forms. As the rim gets thicker, the edge retraction slows down. The rising rim becomes unstable and decomposes resulting in the appearance of two outcomes called pinch-off and fingering instability. The valley behind the rim reaches the surface of the substrate and wire-shaped structure forms, during the pinch-off. Meanwhile, in the fingering process, the rim starts to form finger-like structures of the film. The last part of this evolution is Rayleigh instability which causes the fingers or wire-like structure to decompose into nanoparticles. The properties of NP strongly depend on the annealing temperature, annealing time, the thickness of metallic films, and environment [47]. The modification of NP properties could significantly increase the bactericidal effect similar to topographical features, thus can be used to induce bacterial cell lysis [34,35,48,49]. Therefore, Ti or TiO_2_ thin films with nanostructures could be a good alternative to antibiotics and a new tool to fight a growing issue of antibiotic-resistant bacteria [25,30,50,51,52,53].

Given the importance of biofilm formation in the development and pathogenesis of oral diseases, it is topical to better understand and improve the antimicrobial and anti-biofilm properties of the TiO_2_ thin films decorated with metal nanoparticles. Thus, there is a need for further studies to design better antibacterial biomaterials for long-term applications.

In this study, the deep learning technique for the observation and identification of the presence of bacteria on the surfaces of TiO_2_ coated with nanosilver (Ag) and nanogold (Au) was proposed. This technique enables one to obtain the precise identification and localization of bacteria despite their different sizes, densities, and quality of optical microscope images. The objective of this study is to demonstrate the possibilities of the proposed deep learning technique and to characterize the formed nanoparticles by assessing their antibacterial activity against *Veillonella parvula* (*V. parvula*) and *Neisseria sicca* (*N. sicca*) species. The species were selected according to their possibility to cause dental infections with endocarditis, septicemia, and other severe complications [10,54] as well as because of different respiratory mechanisms (aerobic and anaerobic) which may have unequal resistance properties to nanoparticles. The antibacterial activity of AgNP-coated TiO_2_ showed its potential of reducing the concentration of the bacteria. Such surfaces could be a promising approach for the development of alternative measures used as antimicrobial agents on the surface of implants ensuring a higher and prolonged success rate after implantation procedures.

## 2. Materials and Methods

### 2.1. Formation and Investigation of NP/TiO_2_ Composites

**Figure 1:**
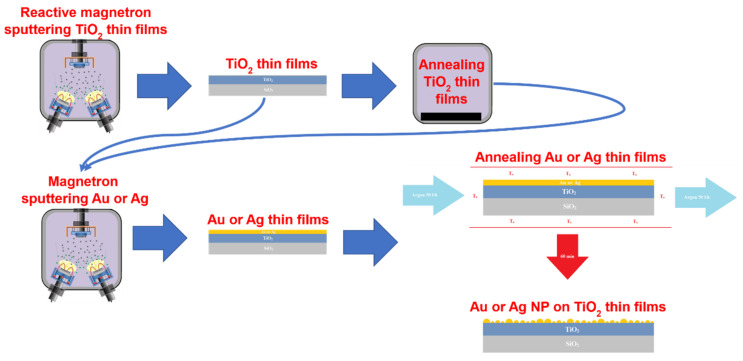
Formation of NP/TiO_2_ structures.

The reactive dual magnetron sputtering method (PVD75 sputtering system, Kurt J. Lesker Company, Pittsburg, PA, USA) and a thermal annealing process (SNOL7/1300LV furnace, Umega, Utena, Lithuania) were used for the preparation of amorphous (Ti20) and crystalline (Ti500) TiO_2_ thin films (Figure 1). The sputtering process was conducted under high vacuum conditions (6.6 × 10^−1^ Pa) in Ar/O_2_ environment. The ratio of the Ar/O_2_ gas mixture was maintained at 8/2. A growth rate of 0.027 nm/s was achieved using two high purity (99.995% purity) Ti targets of 2″ diameter (Kurt J. Lesker Company, Pittsburg, PA, USA) and a constant DC power of 250 W. TiO_2_ thin films (~100 nm) were deposited on rotating (8 RPM) SiO_2_ substrates at room temperature. Obtained thin films were amorphous (Ti20). In order to obtain a crystalline structure (Ti500) and different surface morphology, thin films were annealed at 500 °C temperature in air. The annealing process was composed of heating, temperature holding (soaking), and cooling. Heating and cooling rates were 4 and 2.5 °C/min, respectively, while the soaking time was 10 h. AuNP and AgNP were formed on TiO_2_ thin films of two different types of structures, one of them being amorphous (Ti20) and another one, crystalline (Ti500).

The formation of NP was a two-step process (Figure 1). In the first step, thin Au or Ag film was deposited on the surface of TiO_2_ thin films using 8 RPM rotation speed. The deposition was conducted using the magnetron sputtering technique. Au and Ag were sputtered under high vacuum conditions (2.0 × 10^−1^ Pa) in an Ar gas environment. High-purity (99.995% purity) Au and Ag targets (Kurt J. Lesker Company, Pittsburg, PA, USA) of 2″ diameter were used for the deposition process. The deposition rates of Au and Ag were 0.161 and 0.133 nm/s, respectively, using constant RF power of 50 W. The thicknesses of the nanocrystalline Au and Ag thin films were determined using 5, 7.5, and 10 nm. In the second step which resulted in the formation of the Au and Ag NPs, Au and Ag thin films were annealed at 500 and 400 °C, respectively, in an Ar atmosphere (50 L/h flow rate) for 60 min.

An X-ray diffractometer (XRD) Brucker D8 Discover (Bruker, Billerica, MA, USA) was used to obtain XRD patterns of deposited thin films and nanostructures. The measurements were carried out over the 2θ angle range of 20–70° using CuKα (λ = 0.154059 nm) radiation, 0.01° step, and Lynx eye PSD detector. The peaks in XRD patterns were identified by EVA Search–Match software (Bruker, Billerica, MA, USA) and PDF-2 database. Later on, the crystallite size was calculated using TOPAS 6.0 software (Bruker, Billerica, MA, USA), i.e., the XRD patterns were fitted using the Pawley method and the crystallite size was calculated using the Scherrer equation [55]. The surface topography images were obtained by scanning electron microscope (SEM) Hitachi 132S–3400N (Hitachi High-Technologies Corporation, Tokyo, Japan). Chemical analysis was carried out using an X-ray photoelectron spectrometer (XPS) PHI Versaprobe 5000 (ULVAC-PHI, Chigasaki, Kanagawa, Japan). The following parameters were used: Al Kα (1486.6 eV) radiation, 19.1 W power, 100 μm beam size, 45° measurement angle, 23.50 eV pass energy, and 0.1 eV resolution for detailed chemical analysis. Localized surface plasmon resonance (LSPR) was calculated from absorbance spectra which were measured by the USB4000 spectrophotometer (Ocean Optics Inc., Rochester, NY, USA) in 500–850 nm wavelength range using 0.2 nm step size. Optical microscope (OM) images were taken by a NIKON Eclipse LV100D microscope (Nikon Metrology Inc., Brighton, MI, USA) using 10× magnification. The images of OM were taken in six different places of each sample.

### 2.2. Biofilm Formation Assay

TiO_2_ surfaces coated with Ag or Au nanoparticles were cleaned with Milli-Q water and sterilized with absolute ethanol (Sigma-Aldrich, Burlington, MA, USA). The sterilized surfaces were vertically placed in sterile glass containers filled with 100 mL of Brain Heart Infusion Broth (BHIB, Thermo Fisher, Basingstoke, UK) enriched with 0.5% (*w*/*v*) sucrose (Sigma-Aldrich, Burlington, MA, USA). Cultures of bacterial strains Gram-negative *V. parvula* ATCC^®^ 10790™ and *N. sicca* ATCC^®^ 29256™ were reactivated on Columbia Agar with 5% sheep blood (Thermo Fisher, Basingstoke, UK) at 37 °C under anaerobic (*V. parvula*) and aerobic (*N. sicca*) conditions for 24 h. The concentration of *V. parvula* and *N. sicca* suspension was adjusted to ~1 × 10^6^ colony forming units (CFU)/mL based on the McFarland standard in BHIB and 200 μL of each suspension was added to separate containers containing medium and micro slides coated with Ti surfaces. The containers were incubated at 37 °C aerobically for *N. sicca* and in an anaerobic jar for *V. parvula* for up to 96 h depending on the species ability to form biofilms on TiO_2_ surfaces. Then, bacterial biofilms were fixed using 2.5% (*w*/*v*) glutaraldehyde in 0.05 M sodium cacodylate buffer (Sigma-Aldrich, Burlington, MA, USA) at 4 °C for 2 h followed by fixing with 1% of osmium tetroxide in cacodylate buffer (Sigma-Aldrich, Burlington, MA, USA) for 1 h at 4 °C. The fixed samples were prepared for microscopy by dehydration using ethanol solutions of 25% (*v*/*v*), 50%, 70%, 95%, and 100% for 10 min each.

### 2.3. Observation of Bacteria in an Experimental Optical Microscope (OM) Images

The process of the observation and identification of the presence of bacteria in the acquired experimental OM images was performed in the following steps. Initially, the experimental OM images were preprocessed. Later, training and validation datasets were prepared, and data augmentation techniques were applied to the experimental images. The convolutional neural network (CNN) model was implemented and trained in the following step. Next, the model was used to segment the experimental OM images and to extract bacteria from the background. Finally, the localization of bacteria from the segmented binary representations of experimental OM images was performed and followed by the necessary statistical analysis.

**Figure 2:**
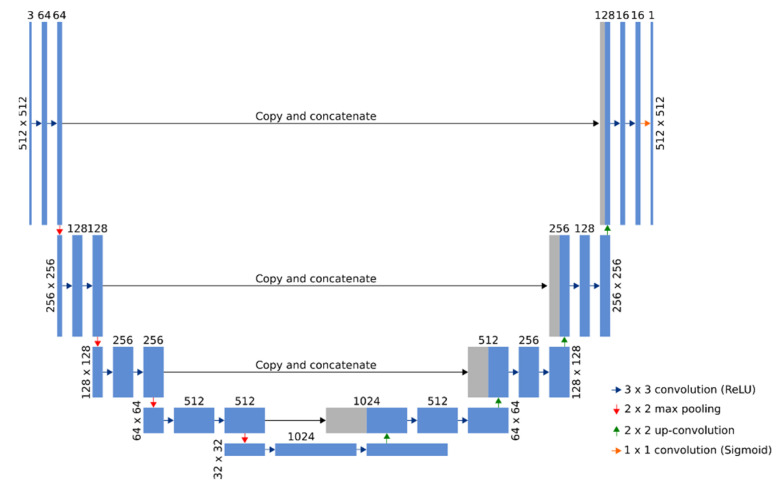
The architecture of a fully convolutional neural network model based on U-Net [57]. Multi-channel feature maps are represented by blue rectangles with dimensions and the number of channels shown on the left and top sides of the rectangle, respectively. The copied feature maps from the contracting path are represented by gray rectangles. The map on the left is the 3-channel patch of the experimental OM image and the map on the right is the binary mask prediction.

The resolution of the acquired experimental OM images was 2560 × 1920. Such images cannot be processed directly as a single dataset due to the large resolution and limited memory on the graphics processing units (GPUs). As a solution to this problem, the original images were padded by 196 and 34 pixels for *x*- and *y*-axes, respectively, using the mirrored pixels of the original image to fill in the missing context. The applied padding procedure allowed us to extract multiple 512 × 512 patches for each experimental OM image, and to employ the overlap-tile strategy for the seamless stitching. These extracted patches were further used for the training, validation steps, and the final segmentation of the experimental OM images. The seamless stitching was employed due to the poor results on the border region obtained with a simple concatenation of the output patches without overlapping. Furthermore, data augmentation (by applying elastic deformations to the patches of experimental OM images) was used to reduce the amount of training data and to allow the network to develop the invariance to deformations [56]. Shear with a range of 0.5, rotation within a range of 180 degrees, zoom with a range of 0.5, and scale with a range of 0.2 were used as the deformation operations. The U-Net [57] deep learning architecture (Figure 2) was used to perform the extraction of images of bacteria from the experimental OM images.

**Figure 3:**
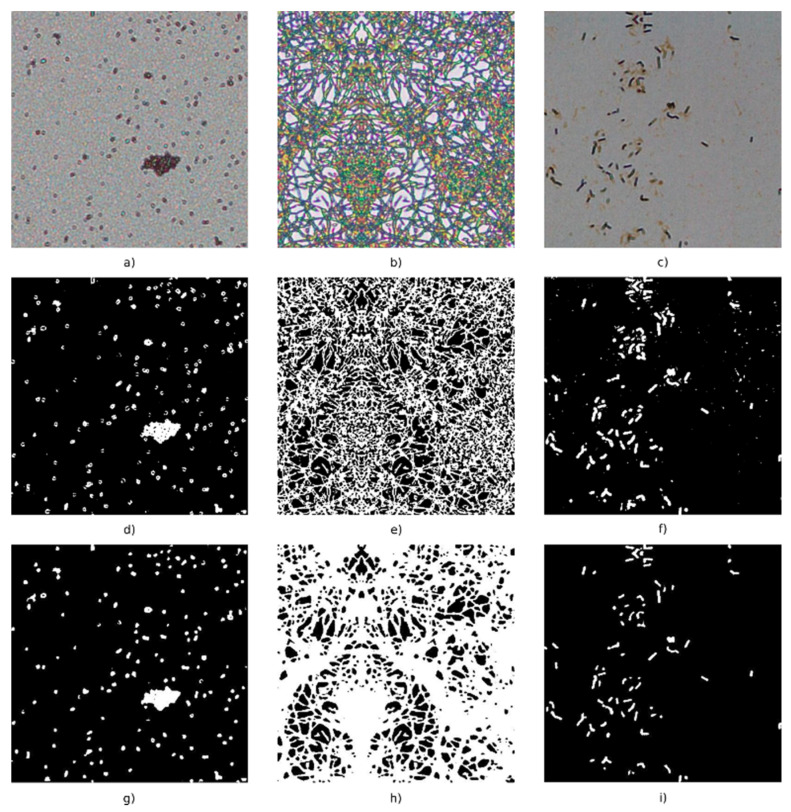
Localization of bacteria attachment and biofilm formation by *N. sicca* in experimental OM images. Acquired experimental images are shown in parts (**a**–**c**). The results of the application of a straightforward binary threshold operation are shown in parts (**d**–**f**). Finally, the results of the application of the fully convolutional neural network model are shown in parts (**g**–**i**).

Adam optimization, a stochastic gradient descent method that is based on adaptive estimation of the first-order and the second-order moments, with the learning rate of 1 × 10^−4^ was used to optimize the model. As only two label classes (background and bacteria) were used, a binary cross-entropy loss function was employed for the computation of the cross-entropy loss between true labels and predicted labels. The loss value of the binary cross-entropy function of the training set was minimized to 0.0516, while the loss value of the validation set was minimized to 0.0568. The model accuracy values of the training set and the validation set were, respectively, equal to 0.9790 and 0.9470. The comparison of ground truth and predicted images showed that most differences between the background and foreground segments appeared on the diffuse borders of the bacterium. The results of the localization of bacteria in experimental OM images are shown in Figure 3. The acquired experimental images are shown in Figure 3a–c parts and their binary representations performed by a straightforward threshold and a fully convolutional neural network are, respectively, shown in parts Figure 3d–f, and Figure 3g–i. The presented results clearly indicate that the applied U-Net model has large advantages over a straightforward threshold operation—the proposed approach is robust to uneven lightning and background noise. That results in more precise identification and localization of bacteria. Furthermore, the results show that this approach is also robust to different sizes, densities, and intensities of bacteria images.

The connected component labeling algorithm [58] was further used to extract and separate individual clusters of bacteria later used for the statistical analysis.

## 3. Results

### 3.1. Surface Characterization

**Figure 4:**
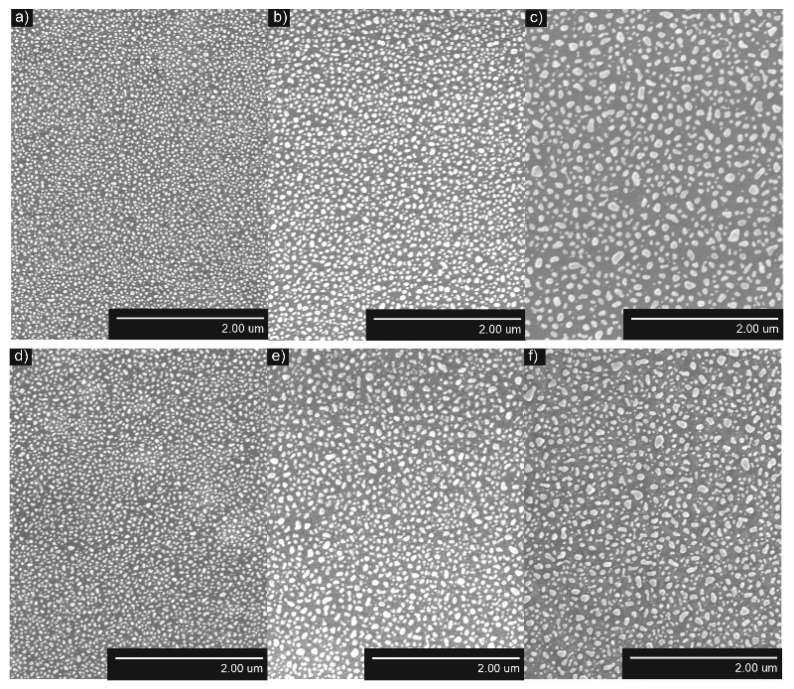
SEM images of AuNP after annealing (*T_a_* = 500 °C) of Au/TiO_2_ thin films system: (**a**) *h* = 5 nm, as-deposited TiO_2_; (**b**) *h* = 7.5 nm, as-deposited TiO_2_; (**c**) *h* = 10 nm, as-deposited TiO_2_; (**d**) *h* = 5 nm, *T*_TiO2_ = 500 °C; (**e**) *h* = 7.5 nm, *T*_TiO2_ = 500 °C; (**f**) *h* = 10 nm, *T*_TiO2_ = 500 °C.

**Figure 5:**
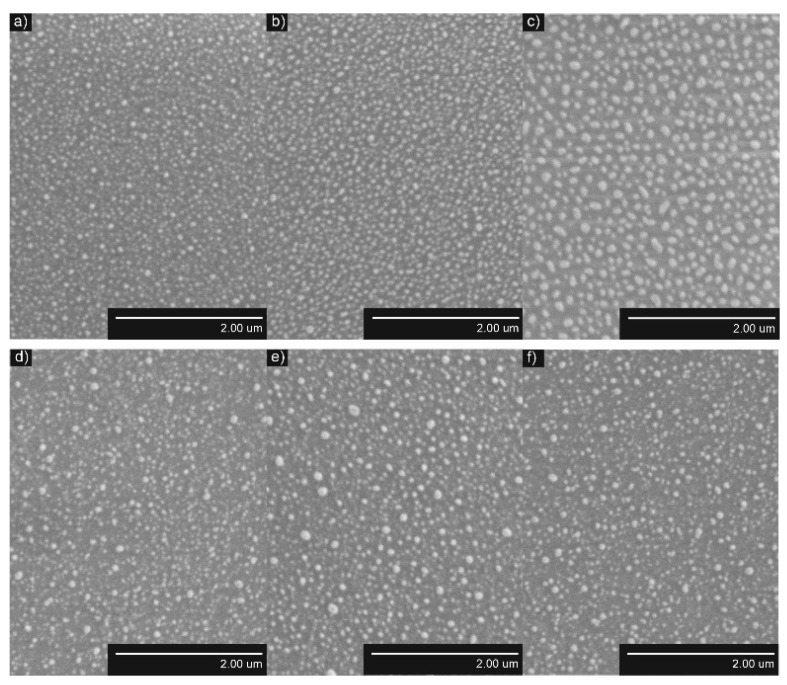
SEM images of AgNP after annealing (*T*_a_ = 400 °C) of Ag/TiO_2_ thin films system: (**a**) *h* = 5 nm, as-deposited TiO_2_; (**b**) *h* = 7.5 nm, as-deposited TiO_2_; (**c**) *h* = 10 nm, as-deposited TiO_2_; (**d**) *h* = 5 nm, *T*_TiO2_ = 500 °C; (**e**) *h* = 7.5 nm, *T*_TiO2_ = 500 °C; (**f**) *h* = 10 nm, *T*_TiO2_ = 500 °C.

The formed AuNP and AgNP were distributed uniformly on the surfaces of TiO_2_ thin films (Figure 4 and Figure 5, respectively). The obtained NP were larger when annealing thicker, thin films, while the density and distance between nearest neighbors were smaller. The structure of TiO_2_ thin films had a minor influence on the density of AuNP and AgNP. The density of AuNP was lower and the density of AgNP was slightly higher in the Ti500 sample group (Table 1).

The quantitative parameters given in Table 1 support SEM images and their interpretation. *D*_mean_ of AuNP increased from 33.3 nm to 59.8 nm for Ti20 group samples and from 37.2 nm to 52.8 nm for the Ti500 group samples when annealing thicker Au films. The same dependence was observed for distances between-NP (border-to-border) to all of their neighbors (*NND_avg_*) of AuNP. It increased from 12.0 nm to 27.5 nm for the Ti20 group samples and from 13.7 nm to 19.3 nm for the Ti500 group samples. The density of AuNP decreased from 316.4 μm^−2^ to 82.8 µm^−2^ for the Ti20 group samples and from 258.8 µm^−2^ to 114.9 µm^−2^ for the Ti500 group samples when annealing thicker Au films. However, the relative area (*SAR*) of AuNP was changing nonlinearly. *SAR* was ~0.30 for the Ti20 group and ~0.32 for the Ti500 group.

Similar results were obtained for AgNP. *D*_mean_ of AgNP increased from 45.2 nm to 86.0 nm for the Ti20 group samples and from 47.7 nm to 50.4 nm for the Ti500 group samples when annealing thicker Ag films. The same dependence was observed for *NND*_avg_ of AgNP. It increased from 31.4 nm to 38.3 nm for the Ti20 group and from 33.7 nm to 37.8 nm for the Ti500 group. The density of AgNP decreased from 85.8 μm^−2^ to 39.2 µm^−2^ for the Ti20 group and from 62.8 µm^−2^ to 54.9 µm^−2^ for the Ti500 group when annealing thicker Ag films. However, the RSA of AgNP was changing nonlinearly. *SAR* was ~0.17 for the Ti20 group and ~0.20 for the Ti500 group.

**Figure 6:**
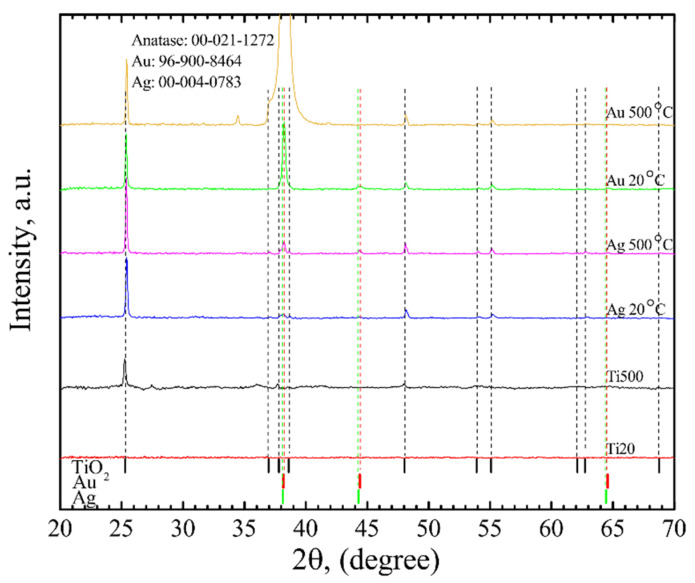
XRD data of AgNP/TiO_2_ and AuNP/TiO_2_ thin film systems.

Most of the NPs were crystalline (Figure 6) and did not experience any phase changes. The crystallite sizes varied from 25 nm to 53 nm for AuNP formed on the Ti20 group thin films and from 27 nm to 84 nm for AuNP formed on Ti500 group thin films. On the other hand, AgNPs were mostly amorphous with the exception of the nanostructures obtained from 10 nm Ag films on Ti500 group thin films. On average, their crystallite size was 60 nm. Moreover, after the dewetting procedure, TiO_2_ thin films belonging to the Ti20 group became polycrystalline with the crystallite size ~110 nm, whereas TiO_2_ thin films belonging to the Ti500 group had the crystallite size of ~129 nm.

**Figure 7:**
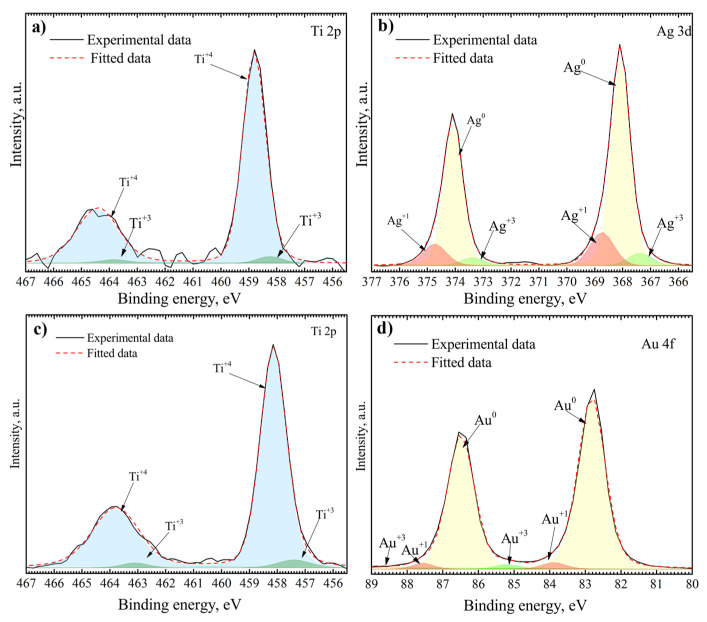
XPS of NP/TiO_2_ system: (**a**) Ti 2p in AgNP/TiO_2_ system, (**b**) Ag 3d in AgNP/TiO_2_ system, (**c**) Ti 2p in AuNP/TiO_2_ system, and (**d**) Au 4f in AuNP/TiO_2_ system.

To better understand surface chemistry, XPS measurements were carried out (Figure 7). The Ti 2p, Ag 3d, and Au 4f spectra were analyzed. The fitting procedure showed that Ti 2p spectra consist of two doublets belonging to Ti^+4^ and Ti^+3^ oxidation states (Figure 7a,c). Ti 2p 3/2 and Ti 2p 1/2 peaks centered at 458.14 eV and 463.84 eV represent Ti^+4^ oxidation state and peaks centered at 457.40 eV and 463.10 eV represent Ti^+3^ oxidation state [59,60,61]. Au^0^, Au^+1^, and Au^+3^ oxidation states were found after the fitting procedure of Au 4f spectrum (Figure 7d). Peaks of Au 4f 7/2 and Au 4f 5/2 components were found at 82.82 and 86.49 eV for Au^0^, 83.89 and 87.56 eV for Au^+1^, and 85.19 and 88.86 eV for Au^+3^ [62]. The Au^0^ oxidation state is the main one with an atomic percentage of ~94%, showing a low amount of gold oxides. The Ag 3d spectrum of AgNP consists of three sets of Ag 3d 5/2 and Ag 3d 3/2 components belonging to Ag^0^, Ag^+1^, and Ag^+3^ oxidation states (Figure 7b). The component positions are 368.07 and 374.07 eV for Ag^0^, 368.74 and 374.74 eV for Ag^+1^, and 367.38 and 373.38 eV for Ag^+3^ [63]. The existence of Ag^+1^ and Ag^+3^ oxidation states shows the presence of various silver oxides on the surface of NP. The calculated atomic percentage of these states is ~17%.

**Figure 8:**
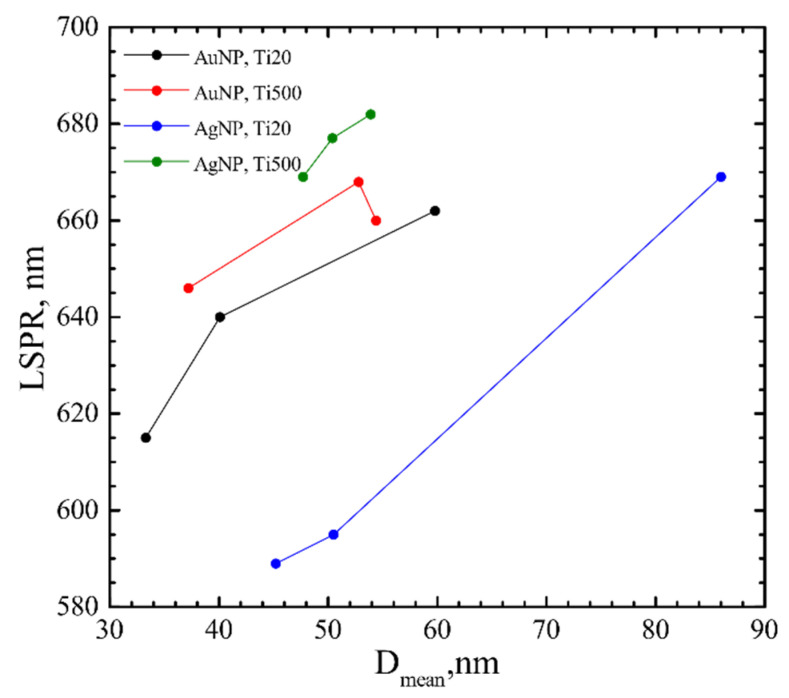
LSPR dependence on mean diameter (*D_mean_*) of AuNP and AgNP on TiO_2_ thin films.

The optical properties were also investigated. LSPR was calculated from the maximum observed in absorbance peak (Figure 8). The LSPR correlated with *D_mean_*, i.e., shifted to longer wavelengths by increasing *D_mean_*. It shifted from 615 nm to 662 nm and from 646 nm to 660 nm for AuNP on Ti20 and Ti500 group samples, respectively, and are similar to other authors’ results [64,65]. Whereas LSPR for AgNP shifted from 589 nm to 669 nm and from 669 nm to 682 nm for Ti20 and Ti500 group samples, respectively. It is shifted to longer wavelengths than found in the literature [66], although the shift could be related to shape effects [67]. Moreover, the LSPR was larger for Ti500 group samples.

### 3.2. Antibacterial Effect

**Figure 9:**
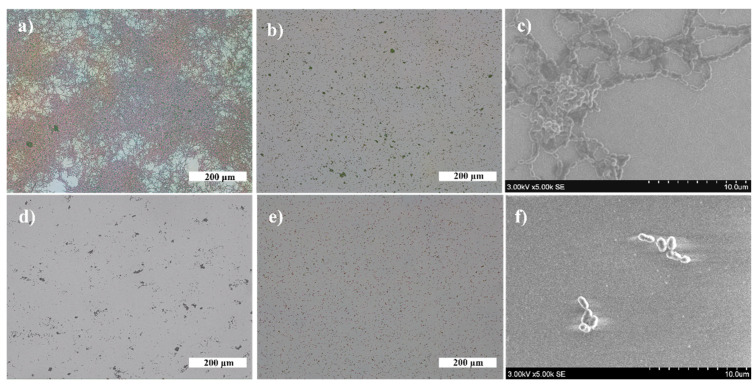
Adherence of bacterial cells on different surfaces (magnification of OM images ×10): (**a**) *N. sicca* on SiO_2_, (**b**) *V. parvula* on SiO_2_, (**c**) SEM image of *N. sicca* on SiO_2_, (**d**) *N. sicca* on TiO_2_, (**e**) *V. parvula* on TiO_2_, and (**f**) SEM image of *V. parvula* on SiO_2_.

The antibacterial properties of SiO_2_, TiO_2_ thin films, and AgNP and AuNP on TiO_2_ surfaces were evaluated using bacterial strains of *V. parvula* and *N. sicca*. First, the growth of bacterial cells was investigated on SiO_2_ (optical glass) and TiO_2_ surfaces to obtain reference samples (Figure 9). A significant difference was found between SiO_2_ and TiO_2_ samples. It was observed that the average size of *Neisseria* cells and their biofilm mass (AS) and surface coverage (SC) are much higher on the SiO_2_ surface than on the TiO_2_ surface: SC is 88.5%, and AS is 347.0 µm for SiO_2_, and SC is 2.7% and AS is 0.9 µm for TiO_2_. Conversely, action on *V. parvula* was different. The bacteria optically looked slightly larger on the SiO_2_ surface than on the TiO_2_ surface (Figure 9b,d). However, the calculations revealed that the AS and SC are almost identical for SiO_2_ and TiO_2_ surfaces (Table 2).

**Figure 10:**
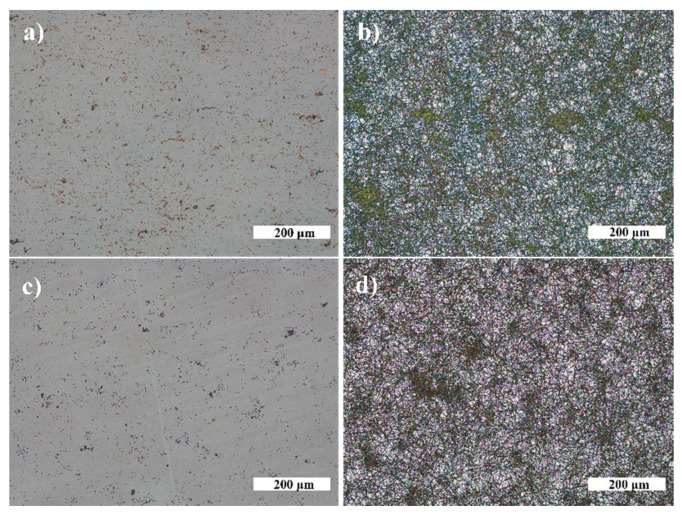
*N. sicca* biofilms on NP (magnification ×10): (**a**) TiO_2_—20 °C, AgNP; (**b**) TiO_2_—20 °C, AuNP; (**c**) TiO_2_—500 °C, AgNP; and (**d**) TiO_2_—500 °C, AuNP.

**Figure 11:**
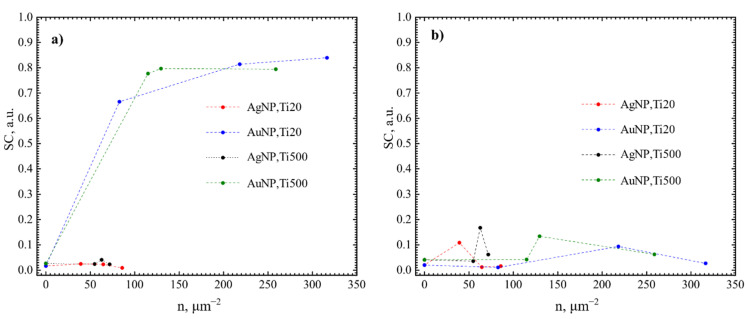
Surface coverage of NP/TiO_2_ system by bacteria: (**a**) *N. sicca*, (**b**) *V. parvula.*

**Figure 12:**
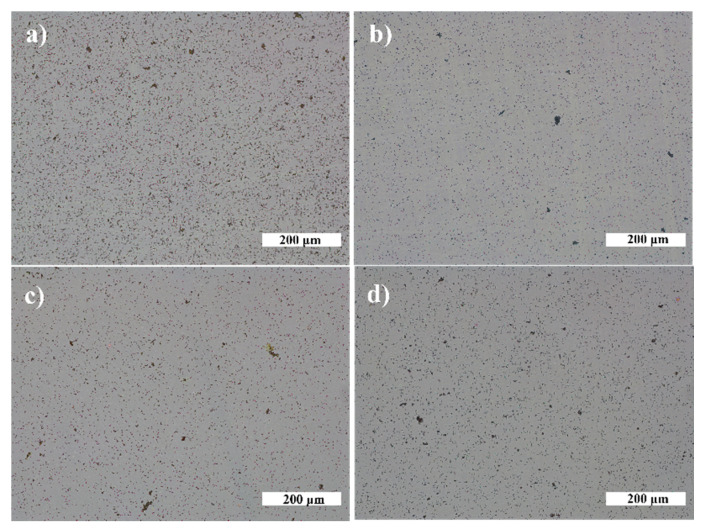
*V. parvula* biofilms on NP (magnification ×10): (**a**) TiO_2_—20 °C, AgNP; (**b**) TiO_2_—20 °C, AuNP; (**c**) TiO_2_—500 °C, AgNP; and (**d**) TiO_2_—500 °C, AuNP.

Next, the antibacterial effect of NP was investigated. Exemplary photos of *N. sicca* colonies on AuNP and AgNP are given in Figure 10. It is obvious that AuNP suppressed the antibacterial effect of TiO_2_. The AS and SC by *N. sicca* on AuNP were similar to SiO_2_ samples (Figure 11a). Bacteria covered 66.6% to 83.4% surface of the sample (Table 2, Figure 12a). On the contrary, the AgNP suppressed the growth of *N. sicca*, and the effect was similar to TiO_2_ thin films (Table 2, Figure 12a). Bacteria covered up to 4.1% surface of the sample. As observed, the bacterial cells on the samples with AgNP are slightly smaller than on bare TiO_2_ thin films. The difference is from 0.1 µm to 0.4 µm. However, the density of the bacteria increased when AS decreased (Table 2). The results demonstrate that the best antibacterial properties against *N. sicca* are shown by the samples with AgNP. The best antibacterial properties were observed for the sample having 85.8 µm^−2^ (Ti20 group) density of AgNP. All bacteria describing parameters (*SC*, *n_B_, AS*, *CV*) had lower values than the group’s average for these samples.

**Figure 13:**
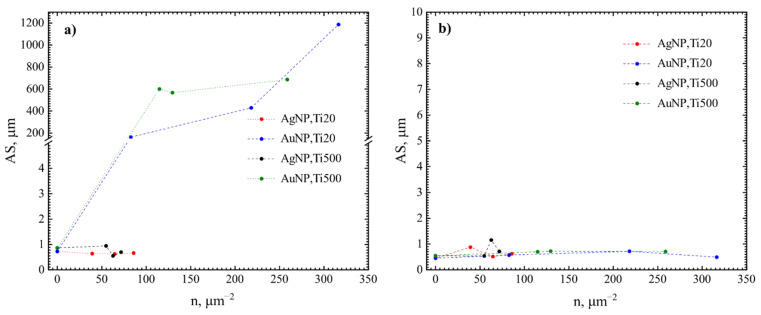
Average size of bacteria colonies: (**a**) *N. sicca* and (**b**) *V. parvula.*

The growth of *V. parvula* on AuNP was significantly different as it was for *N. sicca* bacteria (Figure 11). The AuNP clearly suppressed the growth of *V. parvula*. The SC was up to 9.4% for the Ti20 group of samples, and up to 13.4% for the Ti500 group of samples (Figure 11b and Table 2). The AS was ~0.7 µm for all samples with AuNP (Figure 13b and Table 2). The density of *V. parvula* cells was from 20,251 mm^−2^ to 126,620 mm^−2^ for the Ti20 group and from 59,728 mm^−2^ to 190,816 mm^−2^ for the Ti500 group of samples.

Similar results were obtained for AgNP. The SC was up to 10.8% for the Ti20 group of samples, and up to 16.7% for the Ti500 group of samples (Figure 12b and Table 2). The size of *V. parvula* cells varied between 0.5 µm and 0.9 µm for the Ti20 group of samples, and from 0.5 µm to 1.2 µm for the Ti500 group of samples (Figure 13b and Table 2). The density of *V. parvula* colonies was from 23508 mm^−2^ to 132,131 mm^−2^ for the Ti20 group and from 66,667 mm^−2^ to 141,514 mm^−2^ for the Ti500 group of samples.

The best antibacterial properties are for the samples having 82.8 µm^−2^ density of AuNP and 64.6 µm^−2^ density of AgNP. In comparison, the antibacterial properties describing parameters were very close for 85.8 µm^−2^ density of AgNP sample (Table 2).

In this study, we had demonstrated that TiO_2_ thin films without the NPs on the surface exhibit antibacterial properties against *N. sicca* and *V. parvula* bacteria. The untreated Ti20 thin films exhibit a better antibacterial efficiency than the thin films annealed at 500 °C. Our previous research [68] supports such results. It is known that surface morphology is a very important factor determining the bacteria adhesion to the surface [69]. The previous research showed that the untreated TiO_2_ thin films were of hydrophobic nature with a wetting angle of 90° that resulted in the reduced adhesion of the *N. sicca* and *V. parvula* bacteria, while the annealed TiO_2_ thin films had a wetting angle of 30° and facilitated the bacteria adhesion. On the other hand, Ti500 thin films had a slightly higher roughness, which did not allow the formation of the colonies of the bacteria and allowed only single-bacteria distributions on the surface of thin films. The decoration of TiO_2_ surface by AuNP significantly reduces antibacterial properties of TiO_2_ thin films against *N. sicca* bacteria. On the other hand, decoration by AgNP made no significant difference for antibacterial properties of TiO_2_ thin films although the sample from the Ti20 group with 85.8 µm^−2^ density of AgNP showed slightly better antibacterial properties than bare TiO_2_ thin films. Both Ti20 and Ti500 groups showed antibacterial effect against *V. parvula* bacteria. The effect was similar independently on the type of NP. The best antibacterial properties were found for the samples having ~80 µm^−2^ density of NP from the Ti20 group.

## 4. Discussion

### 4.1. Influence of the Underlying Substrate and Film Thickness on the NP Formation

During the dewetting process, the development of NPs depends on several parameters, e.g., the metal film thickness, annealing temperature, thermodynamic driving force, strain in a thin metallic film, nature and the number of defects, and crystallographic features, etc. [47] In this study, the relation between the film thickness and the size of the formed NP was established. As seen in Figure 4 and Figure 5, after annealing, larger NPs are formed for thicker metallic films. Such kind of dependence appeared due to two reasons [70]. First, a different number of holes formed during the annealing process of thin metallic films depending on their thickness. The number of formed holes is inversely proportional to the thickness. Moreover, very thin films are discontinued and naturally have holes [71]. On the other hand, in the later stages of dewetting, rims break down into wire-like strands through pinch-off or fingering processes. The radii of these strands depend on the thickness of thin films also being larger when thicker films are annealed. The lower number of holes and bigger radii of strands result in the formation of enlarged NPs followed by the lower density of NP and larger distances between NP. Such dependencies correlate with collected data from different studies (Table 3).

As it is seen from Table 3, the underlying substrate, its structure, composition, and surface properties also affect the formation and related parameters of NP. The shape and morphology slightly differ for the NP formed on the different types of substrates. Amorphous substrate Ti20 provides a faster process of dewetting due to the higher interface energy of the Au (Ag)/Ti20 interface. These results are consistent with Nsimama’s findings [80]. AuNPs were crystalline and AgNPs were amorphous in almost all samples. Moreover, amorphous TiO_2_ changed its phase to anatase after the formation of NP, because 400 °C temperature is sufficient for amorphous to anatase transition to occur [81].

The investigation of absorbance spectra revealed the plasmonic nature of Au and Ag NP on TiO_2_ thin films. LSPR values are 615–662 nm for Au NP and 589–682 for Ag NP. It gives the opportunity to use these composite thin films in the biomedical application as antibacterial coatings [82,83].

The release of metal ions and the direct interaction between the surface of NPs and bacteria are among the most investigated antibacterial mechanisms of NPs [84,85]. Therefore, the valence states of the NPs providing the information about the ratio between metallic and oxidized states of the nanoparticles and their influence on the antibacterial effect are usually investigated. The XPS measurements determined Au^0^, Au^+1^, and Au^+3^ oxidation states for gold NP and Ag^0^, Ag^+1^, and Ag^+3^ oxidation states for silver NP. The binding energies of Au and Ag shifted to a lower binding energy state with a decrease in the size of NP and together with the Au^+1^, Au^+3^, Ag^+1^, and Ag^+3^ oxidation states, indicating the presence of Au and Ag oxides at the surface of NPs [63,86]. However, Au^0^ and Ag^0^ are the main oxidation states, indicating a high percentage of metallic gold (94%) and silver (83%) at the surface of NPs. Metallic NPs containing the oxidized states could have beneficial antibacterial effects by combining the metal ion release mechanisms together with the induction of reactive oxygen species (ROS) and possibly other mechanisms which could lead to the enhancement of antibacterial effect [87].

### 4.2. Evaluation of the Antibacterial Activity of the Films Containing Either Au or Ag Nanoparticles

Ag and Au display a well-known broad spectrum of antimicrobial activity, antiseptic effects, and low risk of bacterial resistance [30,88]. Nanoparticles of these elements enhance the antibacterial efficiency due to the size of particles, charge, surface morphology, and structure [89]. Upon interaction with bacteria, NPs bond to the membrane of the bacteria by electrostatic attractions, Van der Walls forces, hydrophobic or receptor–ligand interactions [90]. The size of the nanoparticles allows them to get transferred through the membranes and provokes changes in the membrane’s shape and function by interfering with the metabolic pathways and inhibiting enzymes, deactivating proteins, inducing oxidative stress and electrolyte imbalance, and modifying gene expression levels [90] that consequently result to the death of bacteria. The antibacterial mechanism usually is a consequence of complex and integral mechanisms acting on the bacteria.

Data show that the concentration of oral bacteria species, especially Gram-positive cocci, such as *Streptococcus mutans* and *Streptococcus oralis*, was significantly decreased due to contact with Ag-coated surfaces [91,92]. A similar effect was also detected with some Gram-negative bacteria [93,94] and other types of nanoparticles, such as Au, Cu, and Zn [89]. However, our data suggest a different antibacterial behavior for Gram-negative species of cocci including *N. sicca* and *V. parvula*. The different antibacterial activity may occur due to the bacterial structure of those bacteria and different intrinsic resistance mechanisms. As it is known that *N. sicca* is an oxidase-positive and aerobic species and *V. parvula* is strictly anaerobic bacteria [95,96,97,98], it may be assumed that the mechanism of respiration is not associated with resistance factors against metal nanoparticles and other mechanisms and should be further investigated. It looks as if the metal itself is also an important factor for antibacterial action. Moreover, the data obtained in this study proved that the type of nanoparticles had a substantial influence on the antibacterial efficiency, especially for *N. sicca* bacteria. *N. sicca* tends to form a biofilm when is cultivated on the surface with AuNP and displays a significant decrease in biofilm formation when it is cultivated on the surface with AgNP. A similar tendency is observed for *V. parvula* bacteria as well. The surfaces with AgNPs provide a better antibacterial efficiency compared to the surfaces with AuNPs. Several factors could explain this finding. First, the possible mechanisms of antibacterial activity of Ag and Au nanoparticles differ or depend on the type of bacterium tested, as well on the structure and composition of the bacterial wall [41,94]. AgNPs are known for the antibacterial activity of metallic silver nanoparticles but also the possible release of the biocidal AgNP ions appeared from the nanoparticle surface in an oxygen-containing environment, which later stimulate the oxidative stress and the production of ROS that eventually cause the bacteria cell damage [28,30,99,100]. Yet, the most dominating antibacterial mechanism for AuNPs is related to the simple interaction between the surfaces of NP and bacteria by electrostatic attraction as Au is less reactive than Ag, especially with respect to the oxidation in an oxygen-containing environment, therefore resulting in lower levels of ROS generation [40,101]. Our XPS data confirm the mentioned assumptions and show a higher level of silver ions in Ag^+1^, and Ag^+3^ oxidation states and a higher generation of ROS that is the leading factor of AgNP antibacterial activity compared to the AuNP. Second, the size of the nanoparticles has a supplementary impact on the antibacterial effect. The inhibition effect and stronger antibacterial efficacy on the TiO_2_ surfaces were detected with an increasing density of AuNP and decreasing size of the nanoparticles. It could be explained that higher antimicrobial efficiency correlates with the larger surface area of NP [102].

## 5. Conclusions

A deep learning technique to observe and identify the presence of the bacteria on the surfaces was realized by analyzing the behavior of Gram-negative cocci *Veillonella parvula* and *Neisseria sicca* on TiO_2_ thin films coated with Ag and Au nanoparticles. In addition, the AuNP and AgNP were successfully formed on amorphous and crystalline TiO_2_ thin films by depositing a thin film of silver and gold and annealing it. It was found that the TiO_2_ surface with AgNP exhibited higher antibacterial efficiency and stronger antibacterial activity than Au nanostructured titanium oxide surfaces and effectively reduced the concentration of the bacteria. Additionally, the inhibition effect and stronger antibacterial efficacy on the TiO_2_ surfaces were detected with an increasing density of AuNP. The current study demonstrates the suitability of the deep learning technique as a potential tool to localize the bacteria on the surfaces despite their size and distribution. Moreover, TiO_2_ surfaces coated with AgNP have the potential to be used as a new antibacterial agent against *Veillonella parvula* and *Neisseria sicca*.

## Figures and Tables

**Table 1:** Characteristics of AuNP and AgNP after annealing metal/TiO_2_ thin films systems: surface area ratio (*SAR*), density of NP (*n_NP_*), mean diameter of NP (*D_mean_*), and distances between-NP (border-to-border) to all their neighbors (*NND_avg_*).

*h*, nm	*T*_TiO2_, °C	AuNP				AgNP			
		*SAR*	*n*_NP_, μm^−2^	*D*_mean_, nm	*NND*_avg_, nm	*SAR*	*n*_NP_, μm^−2^	*D*_mean_, nm	*NND*_avg_, nm
10	20 (Ti20)	0.28	82.8	59.8	27.5	0.27	39.2	86.0	38.3
7.5		0.31	218.1	40.1	14.7	0.17	64.6	50.5	40.9
5		0.31	316.4	33.3	12.0	0.17	85.8	45.2	31.4
10	500 (Ti500)	0.32	114.9	52.8	19.3	0.14	54.9	50.4	37.8
7.5		0.34	129.5	54.4	16.5	0.21	71.8	53.9	29.8
5		0.31	258.8	37.2	13.7	0.15	62.8	47.7	33.7

**Table 2:** Parameters describing the growth of *N. sicca* and *V. parvula* bacteria: *n*_NP_—density of NP; *SC*—surface coverage; *n*_B_—density of bacteria cells; *AS*—average size of bacteria colonies; and *CV*—coefficient of variation of clusters size.

*T*_TiO2_, °C	NP	*n*_NP_, μm^−2^	*N. sicca*				*V. parvula*			
			*SC*, %	*n*_B_, mm^−2^	*AS*, µm	*CV*	*SC*, %	*n*_B_, mm^−2^	*AS*, µm	*CV*
20 (Ti20)	Au	0	1.7	23,417	0.7	2.2	2	42,686	0.5	0.9
		82.8	66.6	8017	164.0	20.4	1.1	20,251	0.6	0.8
		218.1	81.4	2375	429.2	11.8	9.4	126,620	0.7	1.5
		316.4	83.4	810	1187.1	6.9	2.7	55,134	0.5	2.1
500 (Ti500)		0	2.7	30,549	0.9	2.0	4.1	75,968	0.5	1.2
		114.9	77.7	1503	600.2	9.4	4.2	59,728	0.7	1.3
		129.5	79.7	1789	567.3	10.2	13.4	190,816	0.7	4.6
		258.8	79.4	1321	687.1	8.9	6.3	89,793	0.7	1.1
20 (Ti20)	Ag	0	1.7	23,417	0.7	2.2	2	42,686	0.5	0.9
		39.2	2.5	49,577	0.6	4.8	10.8	132,131	0.9	2.2
		64.6	2.3	43,801	0.6	3.0	1.2	23,508	0.5	0.8
		85.8	0.9	14,678	0.7	2.3	1.6	25,864	0.6	1.3
500 (Ti500)		0	2.7	30,549	0.9	2.0	4.1	75,968	0.5	1.2
		54.9	2.4	24,874	1.0	1.2	3.6	66,667	0.5	1.4
		62.8	4.1	88,324	0.6	3.0	16.7	141,514	1.2	2.4
		71.8	2.3	45,523	0.7	2.5	6.2	87,496	0.7	1.5
Optical glass		-	88.5	159	347.0	1.4	5.5	96,138	0.6	0.2

**Table 3:** Characteristics found in the literature of AuNP and AgNP after annealing metal thin films: surface area ratio (*SAR*), density of NP (*n_NP_*), mean diameter of NP (*D_mean_*), and distances between-NP (border-to-border) to all their neighbors (*NND_avg_*).

AuNP								AgNP							
*h*, nm	*T*_a_, °C	Substrate	*SAR*	*n*_NP_, μm^−2^	*D*_mean_, nm	*NND*_avg_, nm	Ref.	*h*, nm	*T*_a_, °C	Substrate	*SAR*	*n*_NP_, μm^−2^	*D*_mean_, nm	*NND*_avg_, nm	Ref.
4	900	Sapphire (0001)	-	900	24	36	[72]	5	200	Si	0.41		67	93	[73]
4	400	TiO_2_	0.23	-	36	-	[74]	6	550	Sapphire (0001)	-		25	-	[75]
5	500	Quartz glass	0.65	-	25	-	[66]	10	500	SiO_2_	-		33	-	[76]
7	400	TiO_2_	0.21	-	74	-	[74]	10	550	Sapphire (0001)	-		50	-	[75]
8	900	Sapphire (0001)	-	90	63	98	[72]	12	400	SiO_2_	-		120	-	[77]
10	500	Quartz glass	0.10	-	55	-	[66]	15	600	SiO_2_	-		321	-	[78]
12	900	Sapphire (0001)	-	50	100	150	[72]	20	400	SiO_2_	-		180	140	[79]

## Data Availability

Not applicable.
